# Temperature-dependent virus lifecycle choices may reveal and predict facets of the biology of opportunistic pathogenic bacteria

**DOI:** 10.1038/s41598-018-27716-3

**Published:** 2018-06-25

**Authors:** Halil I. Egilmez, Andrew Yu. Morozov, Martha R. J. Clokie, Jinyu Shan, Andrey Letarov, Edouard E. Galyov

**Affiliations:** 1 Department of Mathematics, University of Leicester, Leicester, LE1 7RH UK; 2 Department of Infection, Immunity and Inflammation, University of Leicester, Leicester, LE1 7RH UK; 3 Winogradsky Institute of Microbiology, RC Biotechnology RAS, Moscow, Russia; 4 Faculty of Biology, Lomonosov Moscow State University, Leninskie Gory, 1, Moscow, 119991 Russia; 5 Moscow Institute of Physics and Technology, Institutskiy per., 9, Dolgoprudny, Moscow Region, 141701 Russia

## Abstract

Melioidosis, a serious illness caused by *Burkholderia pseudomallei*, results in up to 40% fatality in infected patients. The pathogen is found in tropical water and soil. Recent findings demonstrated that bacterial numbers can be regulated by a novel clade of phages that are abundant in soil and water. These phages differentially infect their bacterial hosts causing lysis at high temperatures and lysogeny at lower temperatures. Thus seasonal and daily temperature variations would cause switches in phage-bacteria interactions. We developed mathematical models using realistic parameters to explore the impact of phages on *B. pseudomallei* populations in the surface water of rice fields over time and under seasonally changing environmental conditions. Historical records were used to provide UV radiation levels and temperature for two Thailand provinces. The models predict seasonal variation of phage-free bacterial numbers correlates with the higher risk of melioidosis acquisition during the “warm and wet” season. We find that enrichment of the environment may lead to irregular large amplitude pulses of bacterial numbers that could significantly increase the probability of disease acquisition. Our results suggest that the phages may regulate *B. pseudomallei* populations throughout the seasons, and these data can potentially help improve the melioidosis prevention efforts in Southeast Asia.

## Introduction

Melioidosis, caused by the environmental pathogen *B. pseudomallei*, is a significant and increasingly recognized problem in Southeast Asia and throughout the tropics. It is estimated to cause ~90,000 deaths a year^1–4^. In addition to being a natural pathogen, *B. pseudomallei* has been classified as a potential biothreat agent by the US Centers for Disease Control and Prevention^5^ due to its ability to be aerosolised, severity of the disease and a relatively low infectious dose. Septicaemic melioidosis results in death of ~40% of those who become infected, and it disproportionally affects agricultural workers. Disease control and prevention are problematic. The number of cases is increasing globally, in part due to the global prevalence of diabetes, a major predisposing factor for melioidosis that is also increasing^6^.

In most endemic areas melioidosis is typically seasonal. It is thought that this seasonality can largely be explained by the increased exposure that rice farmers have to the pathogen during the rice planting work that starts at the onset of the monsoon period (for a review see^7^). However, it is also evident that environmental biology of the pathogen is impacted by seasonal factors (e.g. temperature variations), which may significantly influence this seasonality of disease^4^. Despite a substantial research effort and environmental monitoring programmes, many aspects of melioidosis, including the environmental distribution of *B. pseudomallei* and its seasonality and ecology, are still poorly understood^4,8,9^. Specifically, and highly pertinent in this research, the factors that govern bacterial numbers in the soil and surface waters across seasons and geographic regions, are largely unknown.

Recent reports suggest that bacteriophages (phages) are an important factor affecting the existence of *B. pseudomallei* in the environment^10^ and which strangely, has been ignored to date in relation to this pathogen. Phages are bacterial viruses, they are the most abundant biological entity on Earth, and they impact all aspects of bacterial evolution and dynamics. For example, it has been shown that phages (together with nutrient availability) control the length and the amplitude of bacterial blooms in the ocean^11,12^. Phages are likely to significantly impact *B. pseudomallei* numbers in the environment^13^ and the ability of the pathogen to cause the disease. Phage driven *B. pseudomallei* population dynamics are also likely to occur in water or soil, but to date this has not been extensively examined. However, high phage numbers have recently been reported in the soil containing *B. pseudomallei* in natural conditions^8,10,14,15^. It was also shown that the bacterial numbers in the presence of phages can be substantially reduced, affecting the success of bacterial isolation from the soil^10^. However, systematic research into the role of phages in control of *B. pseudomallei* population dynamics is lacking. Determining the impact of phages on *B. pseudomallei* in the environment would therefore improve our understanding and enable forecasting of the environmental dynamics of this important pathogen.

A recent key observation which improves our understanding of *B. pseudomallei* ecology concerns the discovery of an abundant phage type that exhibits ‘condition dependent lysogeny’^8^. In this work, it was shown that one particular phage type was dominant throughout Thailand. The phage follows a lytic cycle in ‘warm’ conditions (so kills the pathogen following infection), but at colder temperatures, remains associated with bacterial cells without causing lysis. As a consequence, it could be expected that in cold conditions most bacterial cells are phage-associated, but these phages would enter the lytic state and lyse their bacterial host following the transition into a warm-blooded host or warmer environmental conditions. These phage-associated bacteria would therefore not be able to cause disease. In contrast, in warm conditions it is likely that the presence of more bacteria in the ‘phage-free’ form (since the numbers of lysogenic bacteria should drop). Such phage-free bacteria therefore have a significant potential to cause disease. The roles of these condition dependent phages during infection were investigated in a recent experiment using a mouse model of infection. Experiments revealed that these phage-associated bacteria are not able to maintain infection^8^.

Mathematical modelling backed up by empirical parameter estimates is known to be an efficient way to understand and predict the dynamics of interactions between phages and their bacterial hosts in various environmental conditions. Computational modelling of bacteria-phage interactions has a long and successful history starting about 60 years ago^16^. Since then a large number of studies have been published in this area exploring various aspects of interactions, such as phage-mediated biodiversity^17,18^, phage-bacteria co-evolution including bacterial resistance^19,20^, control of disease spread such as cholera outbreaks^21^, various applications of phage therapy^21–23^, the influence of environmental factors on control of bacteria by phages^24,25^ or effect of viruses on microbial food webs and ecosystem processes^26^. The use of mathematical modelling generally allows researchers to explore a wide range of parameters under different scenarios of seasonal variation. Surprisingly, no model has been developed yet to assess interaction between phages and *B. pseudomallei* either under laboratory conditions, or in the natural environment.

Here, we build and explore two parsimonious mathematical models to describe and predict daily and seasonal dynamics of the size and composition of *B. pseudomallei* populations controlled by their associated temperature-dependent phages in the surface water, mimicking a top layer of a typical rice field. The main postulate and a concept behind the model are as follows. Environmental populations of *B. pseudomallei* form a predator-prey relationship with a dominant phage type that exhibits temperature-dependent lysogeny. Bacteria can exist in the environment in two forms: susceptible phage-free or infected with the phage. During colder periods of the year, when day and night temperatures stay below 35 °C, bacterial growth is slow, and bacterial populations are increasingly dominated by the lysogens as most of phage infections do not result in bacterial killing. However, during warmer seasons, day time temperatures trigger the phages to go through the lytic cycle, resulting in the killing of the lysogens. At the same time, higher day time temperatures are also permissive for a rapid growth of the remaining susceptible phage-free bacteria; such bacteria become more prevalent in the environment as a result. Thus, the structure of the environmental bacterial population changes during the seasonal progression.

The parameters for our models are taken from either our own experimental research or from relevant scientific publications. As a particular ecological case study, we consider seasonal bacteria dynamics in two endemic regions of Thailand (Sa Kaeo and Nakhon) by using historic data on the temperature variation and the intensity of solar ultraviolet radiation. Our simulation results seem to strongly support the data that show that there is a higher risk of melioidosis acquisition during the “warm and wet” season(s) reported in Southeast Asia^27,28^. This supports and extends the original hypothesis of temperature-dependent phages to be a key regulator of bacterial numbers across seasons. We explore the dependence of bacteria-phage dynamics on the key parameters to be able to explain the observed difference in the disease acquisition rate for different environmental conditions. Our study also emphasizes the roles of the interplay between the variation of temperature and the UV radiation of the seasonal patterns of bacterial numbers.

## Methods

### Developing mathematical models

**Figure 1:**
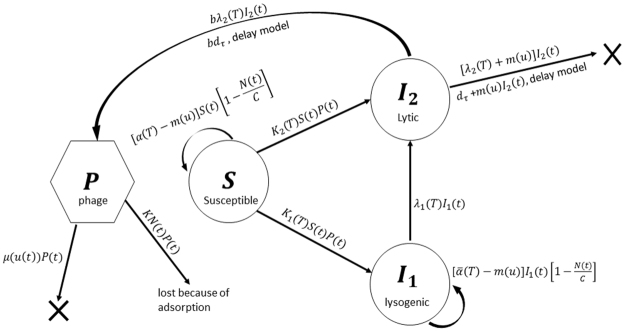
Schematic diagram explaining the bacteria-bacteriophage interactions given by Models I and II. The system consists of four compartments: Susceptible bacteria (*S*), infected bacteria in lysogenic (*I*_1_) and lytic (*I*_2_) state and the free bacteriophages (*P*). Arrows show loses in some compartments or transitions between compartments. The function denoted by *d*_*τ*_ in the figure is $$({K}_{2}(T(t-\tau ))S(t-\tau )P(t-\tau )-$$$${\lambda }_{1}(T(t-\tau )){I}_{1}(t-\tau ))\exp (-{\int }_{t-\tau }^{t}m(u(x)){\rm{dx}})$$.

The aim of our study is to model bacteria-phage interactions in the stagnant water of agricultural fields: we consider the environment to be spatially homogeneous (for simplicity, we have ignored bacteria in the soil). The phage-bacteria system is described by four main compartments: susceptible phage-free bacteria (*S*), infected bacteria in lysogenic (*I*_1_) and lytic states (*I*_2_) as well as free phage (*P*). The flowchart to illustrate this is shown in Fig. 1 and it holds for both models used in this paper. The total density of the host bacterial population is denoted by *N* = *S* + *I*_1_ + *I*_2_. The growth of susceptible phage-free bacteria is described by a standard logistic function parameterisation^25^, where *α*(*T*) is the maximal per capita growth rate; *m*(*u*(*t*)) is the mortality of bacteria due to the exposure to ultraviolet solar radiation^29^ and *C* is the carrying capacity of the environment. This number gives the maximal possible number of bacteria which the environment can sustain. For the purpose of this paper, we assume that the carrying capacity for a given area is constant and accounts for all other (non-phage) factors affecting *B. pseudomallei* existence in the environment. At low temperatures, infection of *S* by the phage results in lysogeny which corresponds to transition from *S* to *I*_1_. Lysogenic bacteria *I*_1_ grow according to the logistic growth function as well but with a different per capita rate $\bar{\alpha }(T)$; the carrying capacity *C* is assumed to be the same for phage-free bacteria. The same concerns the mortality of bacteria due to the UV radiation. At high temperatures, infection of *S* occurs via the lytic scenario which eventually results in cell lysis and death: this is shown by the transition from *S* to *I*_2_. Moreover, an increase in temperature would interrupt the normal lysogenic cycle of *I*_1_ and the infection become lytic: this is described by transition from *I*_1_ to *I*_2_ and is mathematically modelled by the term *λ*_1_(*T*)*I*_1_. The density of free phage *P* in water decreases due to binding to any type of bacteria (described by the term *K NP*) and due to natural mortality *μP*. Note that binding to *I*_1_ and *I*_2_ always result in phage loss. The density *P* increases due to release of new *b* phages as infected bacteria lyse and release phage progeny; *b* is the phage burst size.

There are two major approaches to modelling the replication of phage lysis. The first one is based on the ordinary differential equations framework^25,26,30^ where lysis is modelled by introducing a mortality term *λ*_2_*I*_2_ in the equation of *I*_2_. The mortality rate *λ*_2_ can be estimated based on the average phage replication time; the released number of free phages is given by *bλ*_2_(*T*)*I*_2_. The model equations become

1 $$\begin{array}{rcl}\frac{dS(t)}{dt} & = & [\alpha (T(t))-m(u(t))]S(t)[1-\frac{N(t)}{C}]-{K}_{s}S(t)P(t)\\ \frac{d{I}_{1}(t)}{\,dt} & = & [\bar{\alpha }(T(t))-m(u(t))]{I}_{1}(t)[1-\frac{N(t)}{C}]+{K}_{1}(T(t))S(t)P(t)-{\lambda }_{1}(T(t)){I}_{1}(t)\\ \frac{d{I}_{2}(t)}{dt} & = & {K}_{2}(T(t))S(t)P(t)+{\lambda }_{1}(T(t)){I}_{1}(t)-{\lambda }_{2}{I}_{2}(t)-m(u(t)){I}_{2}(t)\\ \frac{dP(t)}{dt} & = & -KN(t)P(t)-\mu (u(t))P(t)+b{\lambda }_{2}{I}_{2}(t)\end{array}$$

Note that the adsorption of free phages is characterised by the constant *K*, whereas in the infection rate term of susceptible bacteria, the corresponding constant is *K*_*S*_, so we assume that ${K}_{S}={\epsilon }K$ (${\epsilon } < 1$), which indicates that not each binding will eventually result in bacterial infection. Finally, we consider that the mortality rate of *P* depends on solar ultraviolet radiation (UVR) exposure; this quantity is denoted by *u*. The above equations constitute Model I.

The alternative approach for modelling cell lysis and phage replication uses delay differential equations (referred here as Model II). This approach is used somewhat more frequently in the literature^22,25,31–33^. The equations of Model II read as follows

2 $$\begin{array}{rcl}\frac{dS(t)}{dt} & = & [\alpha (T(t))-m(u(t))]S(t)[1-\frac{N(t)}{C}]-{K}_{S}S(t)P(t),\\ \frac{d{I}_{1}(t)}{dt} & = & [\bar{\alpha }(T(t))-m(u(t))]{I}_{1}(t)[1-\frac{N(t)}{C}]\\  &  & +{K}_{1}(T(t))S(t)P(t)-{\lambda }_{1}(T(t)){I}_{1}(t),\\ \frac{d{I}_{2}(t)}{dt} & = & {K}_{2}(T(t))S(t)P(t)+{\lambda }_{1}(T(t)){I}_{1}(t)\\  &  & -{K}_{2}(T(t-\tau ))S(t-\tau )P(t-\tau )\exp (-{\int }_{t-\tau }^{t}m(u(x)){\rm{dx}})\,\\  &  & -{\lambda }_{1}(T(t-\tau )){I}_{1}(t-\tau )\exp (-{\int }_{t-\tau }^{t}m(u(x)){\rm{dx}})-m(u(t)){I}_{2}(t),\\ \frac{dP(t)}{dt} & = & -{K}_{P}N(t)P(t)-\mu (u(t))P(t)+b({\lambda }_{1}(T(t-\tau )){I}_{1}(t-\tau )\\  &  & +{K}_{2}(T(t-\tau ))S(t-\tau )P(t-\tau ))\exp (-{\int }_{t-\tau }^{t}m(u(x)){\rm{dx}}),\end{array}$$

where *τ* is the time between infection and lysis. In the equation for *I*_2_, the first delay term says that those bacterial cells which were infected τ minutes ago are experiencing lysis at the current moment of time. The same concerns the last delay term in equation for *I*_2_ describing the lysis of those former lysogenic cells *I*_1_ which were converted to the lytic cycle τ minutes ago due to an increase of the ambient temperature. The exponential terms take into account the mortality of lytic bacteria due to UV radiation. In the equation for *P*, the mentioned terms are multiplied by the burst size *b* to give the number of released free phages.

Using of each of Models I and II has its advantages and disadvantages for modelling bacteria-phage interaction, thus in this study we will explore both of them. Note also that there exist more complicated bacteria-phage interaction models, for instance explicitly describing the number of phages currently bound to the bacterial cell (susceptible and/or infected) at the current moment of time or some models considering a non-fixed lysis time^25,33^. However, here we intentionally prefer to keep our parsimonious models of temperature dependent lysogeny as simple as possible to be able to understand the generic behaviour of such systems including the dependence of the dynamics on key parameters.

### Model parameterisation

**Figure 2:**
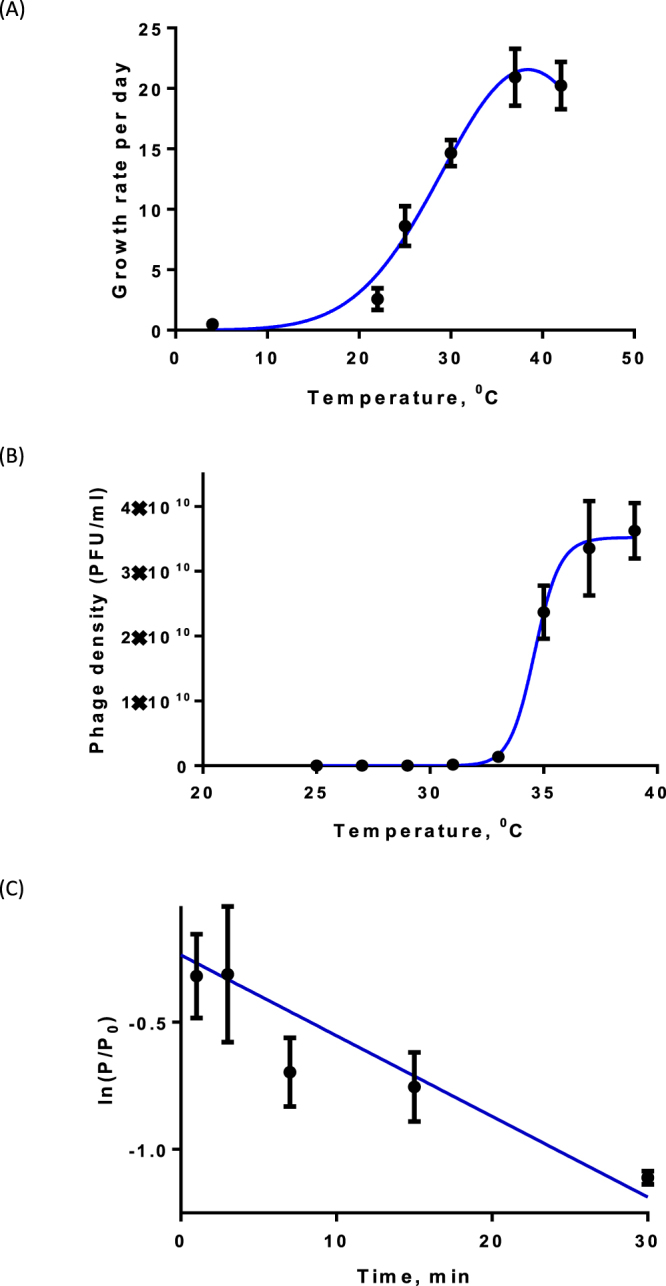
Experimental estimation of the key model parameters. (**A**) Dependence of the growth rate of *B. pseudomallei* on temperature using the experiments of Chen and co-authors^34^. (**B**) Bacteria-phage interactions depending on the temperature (data from this study). The graph shows the density of free phages (see SM1). Appearance of a large number of phages at approximately 34 °C signifies a switch between lysogenic and lytic infection types; (**C**) Binding of phages to bacterial cells (data from this study). The graph shows the natural log of the ratio of the initial number of free phages and the density of phages at time *t* (see SM2). For each experiments, fitting of curves was done using the GraphPad Prism software. For details, see the main text.

The temperature dependence of the bacterial growth rate of *B. pseudomallei* has been evaluated using experimental results of Chen and co-authors^34^. We fitted the average growth rate using 7 strains from the mentioned paper to exclude negative values. We find that the resulting temperature dependence of *α*(*T*) can well approximated by a Gaussian function (see Fig. 2A)

3 $$\alpha (T)=\exp (-\frac{{(T-{T}_{0})}^{2}}{2{\sigma }^{2}}){\alpha }_{{\rm{\max }}}.$$

Our non-linear regression fitting using the GraphPad Prism software gives the following estimates: *σ* = 9.1 + 2.3 °C; *T*_0_ = 38.41 + 1.05 °C; *α*_max_ = 22 ± 1.5 day^−1^ (*R*^2^ = 0.95) Thus the maximal bacterial growth occurs at about 38 °C.

The per capita growth rate of lysogenic bacteria is considered to be given

4 $$\bar{\alpha }(T)=\alpha (T)[\frac{{T}_{1}^{n}}{{T}_{1}^{n}+{T}^{n}}\,]={\alpha }_{{\rm{\max }}}\exp (-\frac{{(T-{T}_{0})}^{2}}{2{\sigma }^{2}})[\frac{{T}_{1}^{n}}{{T}_{1}^{n}+{T}^{n}}].$$

Here the growth rate of lysogenic bacteria at low temperatures is the same as that of susceptible bacteria, whereas at higher temperatures the normal cell division of *I*_1_ stops and bacteria become lytic. The switch between lysogenic and lytic infection scenarios occurs at the critical temperature *T*_1_. We consider that the switching process can be described via a sharp S-shape curve ${T}_{1}^{n}/({T}_{1}^{n}+{T}^{n})$ which - for a large parameter *n* - is close to one for *T* < *T*_1_ and close to zero for *T* > *T*_1_.

The use of the S-shaped curve to describe the lysogenic-lytic switch is confirmed by our experimental results presented in Fig. 2B. We used a standard plaque assay to assess lytic-lysogenic transition rates at different temperatures. Briefly, *B. thailandensis* E264 was infected with ΦBp-AMP1 at an MOI of 1 at room temperature for 15 min. Serial dilutions of the infection mixture were then spotted onto identical plates of confluently grown *B. thailandensis*. The plates were then incubated at different temperatures for appropriate time periods, and the number of plaques was counted to estimate number of productive lytic infections that occurred at each incubation temperature. More details on experimental work are given in Supplementary Material SM1. These data clearly show that the number of plaques is very low for 25 °C < *T* < 32 °C which clearly demonstrates that most phage-bacteria interactions result in lysogeny. For higher temperatures *T* > 34 °C the number of plaques produced as a result of infection of susceptible bacteria increased sharply. This means that most of the viral infections follow the lytic cycle. Our fitting of the S-shaped curve gives the following estimates *T*_1_ = 34.60.02 °C and *n* = 55 ± 7 (*R*^2^ = 0.97). Thus the switch between lysogenic and lytic infections occurs at approximately 35 °C, which is an important biological finding for temperature-dependent lysogeny of *B. pseudomallei*. The results of this experiment show that the temperature-dependent lysogeny is a gradual process, i.e. at *T* = *T*_1_ approximately 50% bacteria are lysogenic and 50% still follow the lytic cycle. Using this experimental result, we can parameterise the dependence of the adsorption on the temperature as

5 $${K}_{1}(T)=(\frac{{T}_{1}^{n}}{{T}_{1}^{n}+{T}^{n}}){K}_{s},$$

6 $${K}_{2}(T)=(\frac{{T}^{n}}{{T}_{1}^{n}+{T}^{n}}){K}_{s},$$

where *K*_*S*_ is the phage adsorption constant, which we assume to be temperature independent. Note that the sum of *K*_1_ and *K*_2_ equals *K*_*S*_. Using the same temperature dependence for the switch rate *λ*_1_ between lysogenic and lytic state we obtain

7 $$\begin{array}{c}{\lambda }_{1}(T)\end{array}=(\frac{{T}^{n}}{{T}_{1}^{n}+{T}^{n}}){\lambda }_{{1}_{\max }},$$

where the maximal transition rate ${\lambda }_{{1}_{\max }}$ is assumed to be equal to the maximal growth rate of the bacteria.

The overall adsorption constant *K* of phages (${K}_{S}={\epsilon }K$) was estimated from our experiment represented in Fig. 2C. We plot the natural logarithmic ratio between the initial number of phages at the start of the experiment and the number of phages at time *t*. The details on the experimental procedure is provided in Supplementary Material SM2. The fitting of the points with a straight line gives *K* = 30.7 ± 5.7 × 10^−8^ ml^−1^day^−1^ (*R*^2^ = 0.84). This value is within the reported values from the literature^35^; however, since other sources report a wide range of *K*, we will vary its value to reveal dependence of model outcome on this parameter.

The other model parameters are taken from the literature. In particular, the burst size of phage *b* is estimated^14^ to vary within the range of 100 and 212. We consider it to be temperature-independent. The value of carrying capacity *C* of *B. pseudomallei* in rice fields is not well-known since different sources provide different estimates^9,36,37^. It is also likely that *C* highly varies from field to field since it largely depend on pH, iron concentration, fertilisers, carbon/nitrogen ratio^9^. We consider the default value of *C* = 2 × 10^6^cell/ml^−1^, which is located in the middle of the reported values. However, we also explore a wide range of *C* to be able to model nutrient poor and nutrient rich environment and the effect of fertilises. The lysis rate of bacteria *λ*_2_ in Model I is assumed to be constant. Since the average latent period of infection is approximately *τ* = 50 min^14^, we may assume that after 50 min 50% lytic on average die, this gives an estimate for *λ*_2_ to be 20 day/ml. The phage infection efficiency coefficient *ε* is not well known; here we assume it to vary within 0.2–0.6, which is in the line with other estimations^38^.

The mortality of free phages *μ* is a key parameter of the model. Such mortality can be caused by exposure to ultraviolet (UV) solar radiation^29^, adsorption to particles other than bacterial cells, consumption by flagellates or amoebas^39^. In this paper, we consider the phage mortality to be

8 $$\mu (u)=\{\begin{array}{cc}{\mu }_{c}+{Y}_{0}\,\exp (ku)\, & {\rm{daytime}}\\ {\mu }_{c} & {\rm{at}}\,{\rm{night}}\end{array},$$

where *μ*_*c*_ is the background (light-independent) mortality; *u* is ultraviolet (UV) index which is measured in units from 1–12. The choice of the exponential dependence on *u* is our modelling choice; however, considering other dependences (e.g. a linear function) do not largely affect model predictions. We estimated the parameters *Y*_0_ and *k* from the phage mortality data in summer and winter in tropical regions^29^. In particular, it is reported that phages suffer 90–95% mortality over a day due to exposure to sun radiation in summer and 50% in winter. Our calculations (see Supplementary Material SM3) give the following estimates for *Y*_0_ = 0.0746 day^−1^ and *k* = 0.366. The background mortality parameter *μ*_*c*_ is extremely hard to estimate (e.g. it may depends on the abundance of flagellates), so we consider the default value of *μ*_*c*_ = 0.1 day^−1^. We also vary this parameter to check the sensitivity of model predictions to *μ*_*c*_. For bacterial mortality caused by the exposure to UV radiation we assume that *m*(*u*) = *Y*_0_exp(*ku*). In this paper, we apply modelling to describe bacteria-phages interactions in two important provinces of Thailand: Nakhon Phanom and Sa Kaeo, and we use the average historical data on UV radiation from the website^40^. Variation of UV index across the year in the considered areas is shown in the Supplementary Material SM4 (Fig. S1). We take a monthly average of the UV index and use interpolation to describe UV variation each day of the year. We also take into account the variation of the length of day time and night across the year to calculate the exposure of phages to UV. For this purpose we used sunset-sunrise time reports from the website^41^.

**Figure 3:**
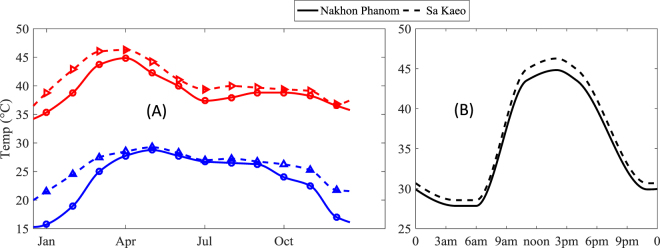
(**A**) Seasonal variation of the maximal and minimal surface temperatures in two endemic provinces in Thailand: Nakhon Phanom and Sa Kaeo. (**B**) Hourly temperature values within day of April 1^st^ (2016) for the same provinces in Thailand. The temperatures are averaged over 3 years: 2013–2016. The data is obtained from the website www.worldweatheronline.com.

Finally, to parameterise Models I, II we consider daily and seasonal variation of temperatures in the mentioned provinces of Sa Kaeo and Nakhon in Thailand. Using the information on historical temperatures from the website^42^, we computed the 4 year average (2013–2016) of the mean monthly temperatures of the air. To obtain the highest surface temperature, we multiply the maximal temperatures by an empirical coefficient η = 1.15 which allows to provide realistic surface temperatures. The temperature variation across the year is found by piecewise cubic spline interpolation of monthly average values for two provinces (see Fig. 3A). The daily temperature variation for each day was approximated using the minimal and the maximal temperatures in Fig. 3A and shape temperature variation at the start of each month (for the other days of the month the shape of the temperature variation was considered to be the same). An example of daily temperature variation on April 1^st^ 2016 is shown in Fig. 3B for both Sa Kaeo and Nakhon; one can see the shapes of the curves are close to each other.

The unit of the densities of bacteria and phages are taken as cell/ml. The summary of model parameters as well their values are provided in Table 1. The datasets generated during and/or analyzed during the current study are available from the corresponding author on reasonable request.

**Table 1:** Parameters used in Models I and II along with their units and ranges.

Symbol	Meaning	Unit	Range	Default value
*α*_max_	Maximum growth rate of bacteria	day^−1^	19–27	23
*C*	Carrying capacity of bacteria	m1^−1^	2 × 10^6^	2 × 10^6^
*K*	Phage adsorption rate	m1/day		10^−7^
*K*_*s*_	Effective per bacteria contact rate	m1/day	—	${\epsilon }\,{K}_{P}$
$\epsilon$	Adsorption efficiency	—	—	0.3
${{\lambda }}_{{1}_{\max }}$	Maximum lysogenic process rate	day^−1^	19.1–27.2	23
*λ*_2_	Constant lysis rate	day^−1^	20	20
*b*	Virus replication factor	—	158 ± 54	100
*T*_0_	Optimum temperature forgrowth and lysis	°C	35.6–50.6	38.2
*T*_1_	Transition temperature	°C	34.81–34.84	34.8
*σ*	Growth rate constant	°C	6.7–17.4	9.1
*u*	Ultraviolet index	—	8–12	—
*μ*_*c*_	Constant mortality rate with UV	day^−1^	—	0.1
*n*	Transition width	—	53.7–56.3	55

## Results

**Figure 4:**
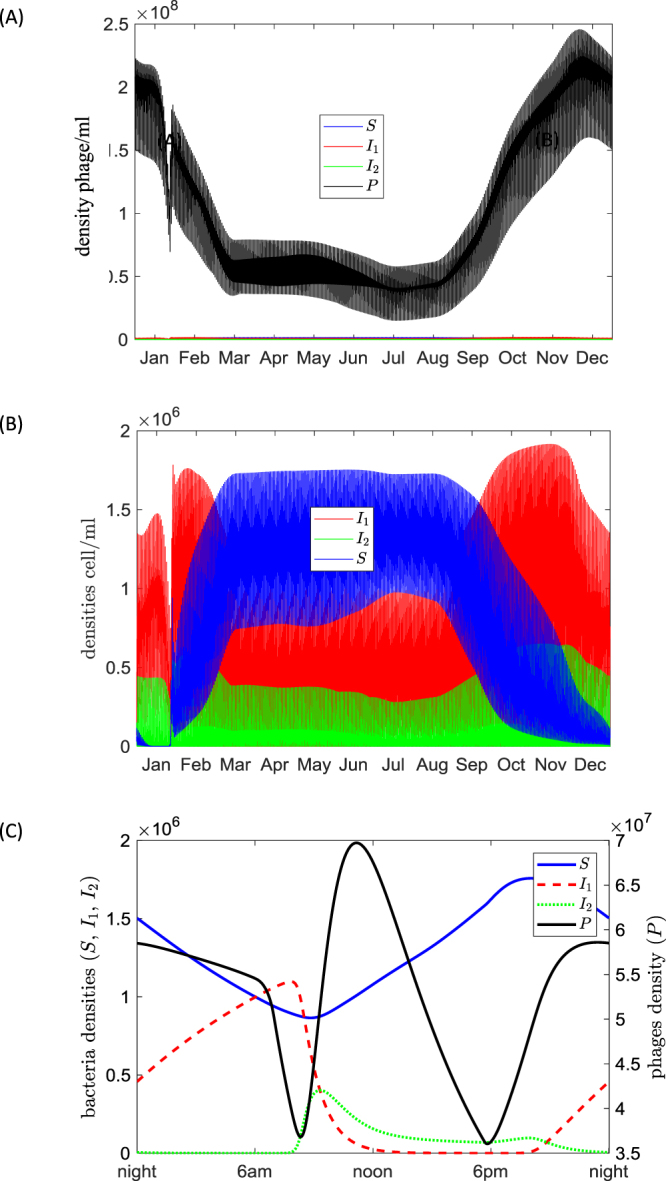
Seasonal and daily dynamics of the bacteria-phage system predicted by Model I for the temperature and solar radiation records corresponding to the Nakhon province. (**A**) Seasonal variation of free phage density. (**B**) Seasonal variation of free-phage bacteria (blue curve), lysogenic bacteria (red curve) and bacteria in the lytic state (green curve). (**C**) Daily variation of bacterial (coloured curves) and phage (black curve) densities on April 1^st^. The model parameters are taken from Table 1 as default values. The unit of the densities of bacteria and phages are cell/ml and phage/ml, respectively.

A typical pattern of the dynamics of bacteria-phage interactions during the year is shown in Fig. 4A,B. Here we exclude transient dynamics that occur within first 1–2 year time frame. The curves in the figure are based on Model 1 and are constructed for the temperature and UV variation corresponding to the Nakhon province. The model predicts a clear seasonal variation of the density of free phages and susceptible bacteria. In particular, values of *P* are lower during the period of March-September and are higher in winter. The numbers of *S*, phage-free bacteria which present major danger for humans, are higher in spring and summer. Interestingly, the number of bacteria *I*_2_ in lytic stage is fairly constant throughout the year. This is possible since the ranges of daily temperature variation allows for both lytic and lysogenic types of infections each day of the year (see Fig. 3A). The observed permanent presence of *I*_2_ explains the fact that free phages *P* can persist across the whole year since their numbers always replenishes through lysis of *I*_2_.

The numbers of all four modelled biological entities exhibit high amplitude daily oscillations. Figure 4C shows dynamics of bacterial and phage densities on April 1^st^ during the warmest period of the year in Thailand. Our model suggests that the dominance of lysogenic and lytic bacteria is highly variable across the day: at night phage infections mostly result in lysogenisation of bacteria, whereas day time infections are mostly lytic. The density of susceptible phage-free bacteria is maximal in the evening (around 8 pm), and it is minimal in the morning (around 9 am).

**Figure 5:**
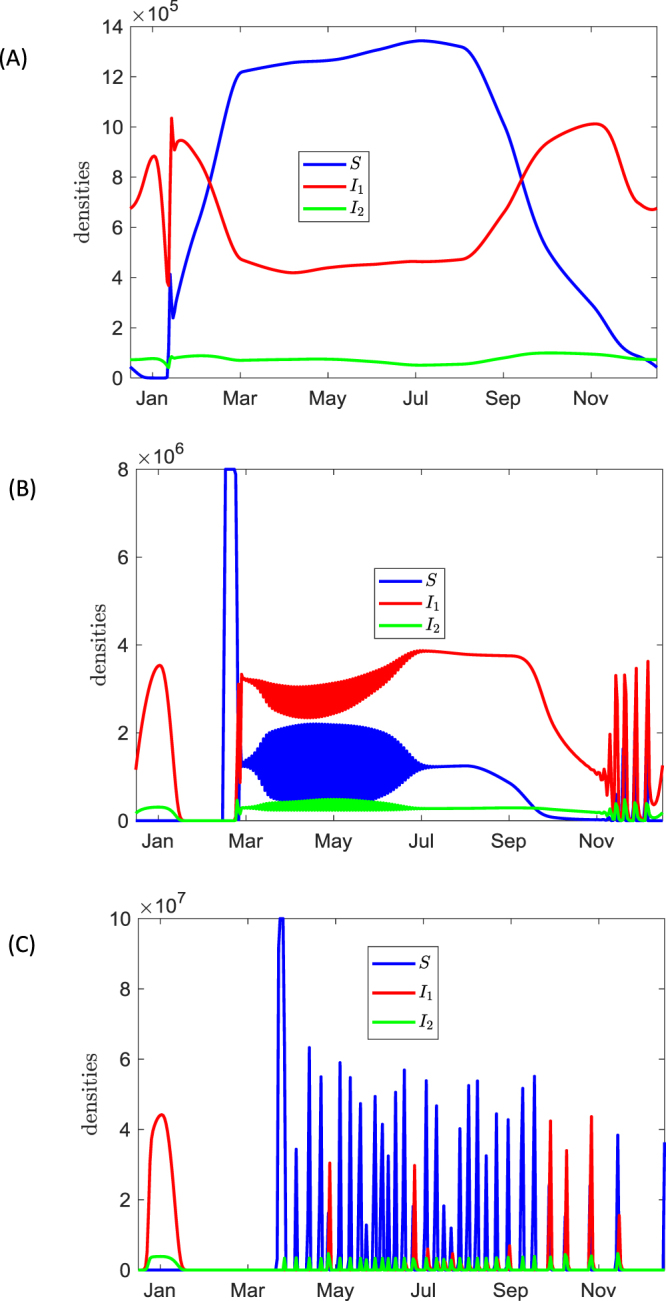
Variation of daily average densities of bacteria across the year for different values of the carrying capacity *C* (Nakhon province): (**A**) *C* = 2 × 10^6^ cell/ml (**B**) *C* = 8 × 10^6^ cell/ml, (**C**) *C* = 1 × 10^8^ cell/ml. Simulations are based on Model I, the other parameters are taken from Table 1 as default values.

To reduce the complexity caused by high frequency daily oscillations, we plot the seasonal variation of daily averaged densities of *S*, *I*_1_ and *I*_2_. This is shown in Fig. 5A. Now it can be clearly seen that seasonal blooms of phage-free bacteria with highest numbers of *S* occur in March-September. The increased abundance of the pathogen should positively co-vary with the risk of disease acquisition.

We also investigated the bacterial-phages dynamics for temperature and solar variation corresponding to the Sa Kaeo province. The results are presented in the Supplementary Material SM5 (Figs S2 and S5A,B). Overall, we find that the model predicts similar daily and seasonal variations of species densities although the densities of *S* are higher in winter season. Interestingly, implementation of Model II (based on delay equations) provides similar predictions about seasonal and daily variation of bacteria and phage densities in Nakhon for the same model parameters. The corresponding graphs are shown in the Supplementary Material (SM5, Fig. S3). In particular, the numbers of susceptible bacteria *S* are higher during warm season whereas the phage numbers are higher during colder seasons.

It is of practical interest to investigate the dependence of bacteria-phage dynamics on the nutrient status of the environment. For instance, bacteria populations can be largely affected by nutrient enrichment via the use of fertilises. This is described by the parameter *C*. The outcomes of variation of the carrying capacity on bacteria-phage interaction are shown in Fig. 5 whereas Fig. S5 from SM5 demonstrates the corresponding dynamics of the phage density. Note that similar dependence of bacterial population dynamics on the carrying capacity is observed for the temperature variations corresponding to the Sa Kaeo province (see SM5, Fig. S5).

A 4-fold increase of *C* compared to the default value would result in the appearance of a large outbreak of *S* in March (see Fig. 5B). The bacterial density in this outbreak is close to *C*. This month-long outbreak is followed by almost periodical oscillations of species densities until early July. Note that unlike in Fig. 4, pronounced oscillations in bacterial densities in Fig. 5B are not daily periodic oscillations but have a period of two days (see SM5, Fig. S6). Another pattern of species density oscillations is observed in late autumn; however, it is characterized by a smaller amplitude of *S*. A further increase in *C* leads to a more irregular dynamics shown in Fig. 5C. The outbreaks of *S* occur within March-July with less frequent peaks in autumn. The small period of high species densities are separated by periods where the density is very low (>10^3^ cells/ml). Our simulations show that fixing the temperature constant (and equal to its average value) in summer still allows long term oscillations which is a characteristic behaviour for ‘classical’ predator-prey cycles in ecosystems under eutrophication^43^. However, for high values of *C* Model II predicts the existence of irregular oscillations throughout the whole year with only a slight degree of seasonality.

**Figure 6:**
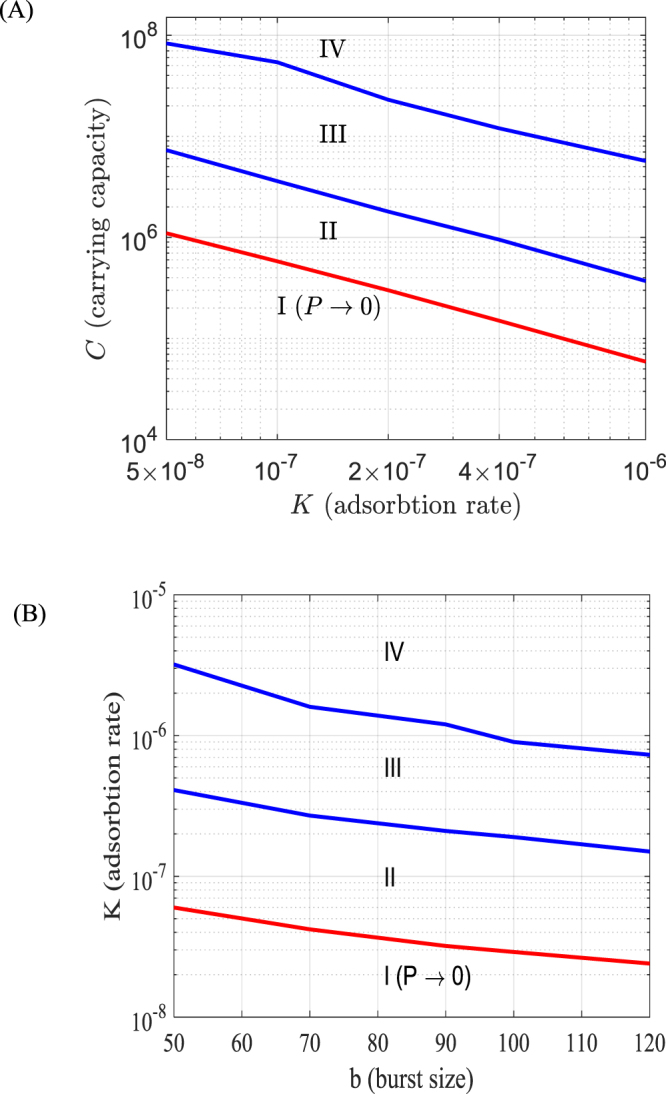
Bifurcation diagrams showing possible dynamical regimes in Model I (Nakhon province) depending on the parameters *K* (overall phage adsorption rate); *C* (carrying capacity of bacteria) and *b* (burst size of phages). The curves in the diagram demark regions with different dynamical patterns: domain I signifies extinction of phages; domains II–IV correspond to the co-existence of bacteria and phages via patterns of dynamics shown, respectively in Fig. 5A,B,C. The red curve is the boundary of the parameter domain where the phage population goes extinct. Other parameters are taken from Table 1 as default values.

We thoroughly explored the dependence of dynamical regimes on the combination of key parameters *C*, *b* and *K*. The results are presented in the form of bifurcation diagrams shown in Fig. 6. In these diagrams the dynamical regimes are: regime (I) signifies extinction of phages in the system and only phage-free bacteria *S* can survive; regimes (II–IV) corresponds to the patterns of dynamics shown in Fig. 5A,B,C, respectively. In regimes (II–IV) all four compartments coexist through the year. Note that it is hard to trace the exact boundary between (II, III) and (IV). Our classification of regime (III) is based on the requirement that there should be species oscillations in summer with a period >1 day with species densities staying below a certain threshold (here we use *S*_0_ = 10^3^ cells/ml as a threshold), whereas for regime IV the minimal density of *S* through oscillations in summer should be smaller than a certain threshold. The data suggest that a decrease in the carrying capacity of bacteria or the adsorption rate of phages would result in phage extinction, whereas high values of these parameters will cause irregular oscillations of species densities. Increasing of burst size would result in persistence of phages; however, it has less pronounced effect on the system dynamics as compared to the other parameters. Finally, we find that variation of *μ*_c,_ the background mortality of phages, within the region of 0.1–0.5 day^−1^ does not affect population dynamics significantly.

We explore the mechanisms controlling the dynamical patterns observed in the models. We check the separate impact of variation of the level of UV intensity (given by *u*) across the year and that of the ambient temperature *T*. We firstly artificially keep a low and a high temperatures to be constant and only vary *u* following the historic data. We find that for the default value of the carrying capacity *C* = 2 × 10^6^ cell/ml and high temperatures the seasonal dynamics of bacterial numbers remains similar as in the complete model (Fig. S7). This signifies that the phages are mainly controlled by the solar radiation and their high mortality during the period of March-September is caused by a high *u*, thus *S* would increase during that period due to reduction of free phage numbers. This is supported by the fact that in simulations with a seasonal variation of temperature but a constant *u*, a pronounced long-term dominance by phage-free bacteria *S* during hot seasons is not observed (however, some seasonality patterns with short peaks of *S* can be still seen, see Figs S10–S12). On the other hand, at lower temperatures, variation of *u* does not result in seasonality in *S* (Fig. S8), even in the case where *T* is equal to the average year temperature (*T* = 29.1 °C) (Fig. S9). Thus the seasonal pattern observed in the complete model (Fig. 5A) is the result of the interplay between both the temperature and the UV level variations.

In more eutrophic environment (e.g. *C* = 1 × 10^8^ cell/ml), keeping the UV intensity constant only has a weak effect on model dynamics, whereas variation of temperature is crucial for observing seasonal patterns (the corresponding figures are not shown for brevity). This signifies that in a nutrient rich environment the driving force is host-pathogen (internal) interaction which is promoted by the seasonal variation of daily temperature ranges: during warm seasons a large part of the day would correspond to lytic infection cycle with a release of a large number of free phages. Interestingly, removing the UV-caused mortality of bacteria in the model does not significantly affect the population dynamics of both bacteria and phages. This can be explained by the fact that the main source of mortality of bacteria is their infection by phages rather than the solar radiation, especially during periods of high temperatures where the intrinsic bacterial growth largely dominates the UV-caused mortality rate. The above results hold for both models I,II.

## Discussion

Melioidosis caused by the bacterial pathogen *B. pseudomallei* is now well-recognised as a serious health threat in Southeast Asia and other areas of endemicity^1,3,4^. Numerous studies have shown that the pathogen is commonly found in soils and stagnant waters throughout these areas^34,44,45^. The novel dimension in explaining population dynamics of this pathogen is built upon recent discoveries of the roles bacteriophages have in controlling bacterial numbers in the environment. The influence of phages on the population dynamics of some bacterial pathogens is recognised and well documented. For example, the prevalence of environmental phages infecting *Vibrio cholerae* has been shown to inversely correlate with seasonal epidemics of cholera^46^, suggesting that phages may have an impact on the seasonality of bacterial infections. Surprisingly phages have been almost ignored within the literature on the environmental biology of *B. pseudomallei*, and the goal of this paper is to bridge this gap. An important particularity of interactions between *B. pseudomallei* and the dominant clade of environmental phages is the phenomenon of condition dependent lysogeny: the type of infection (lytic or lysogenic) would be determined by the ambient temperature^8^. Seasonally oscillating levels of UV solar radiation also strongly affect the mortality of phages and bacteria, making the system even more complex^29^. In this case, mathematical modelling is a natural tool for improving our understanding of the main features of seasonal dynamics of *B. pseudomallei*. To the best of our knowledge, the models considered here are the first to describe *B. pseudomallei*-phage interactions in an attempt to predict seasonal variations in the structure of the bacterial population in the environment.

Our models are based on realistic estimates of biological parameters and on the historical data on temperature as well as solar radiation. Our experimental results on the temperature dependent lysogeny demonstrated that the switch between lysogenic and lytic way of infection occurs at around *T*_1_ = 35 °C (see Fig. 2A) which was previously underestimated in the literature^8^. Our investigation of the typical ranges of temperature variation in two provinces of Thailand (e.g. Fig. 3) strengthens our early hypothesis that the temperature dependent switch between lytic and lysogenic infections would be observed in most of days during year. We also find that the estimated value of the adsorption constant of *B. pseudomallei* phages (which was earlier an unknown parameter) allows the persistence of phages in the models.

Our model simulation predicts high variations of *B. pseudomallei* and phages numbers both daily and seasonally. Note that without the phage component, the same model would predict a constant bacterial density throughout the day and across seasons which would be close to the carrying capacity *C* of the environment for *B. pseudomallei*. We find that elevated numbers of susceptible phage-free bacteria *S* are observed in warm seasons with a high solar radiation. The model predicts that *B. pseudomallei* undergo an annual “invisible bloom” during these seasons. We suggest that the elevated densities of phage-free bacteria would indicate higher infection risk for agricultural workers, and this correlates well with the seasonality of reported cases of disease acquisition in some regions in Thailand such as Ratchatani^27^ or Nakhon Phanom^28^. On the other hand, there is no pronounced annual variation of infection cases in Sa Kaeo^43^ as it is predicted by the model. Such a discrepancy can potentially be explained by different environmental conditions in terms of soil properties (resulting in different carrying capacity *C*) which would dampen the seasonal variation in the system. On the other hand, the relation between the density of phage-free bacteria and disease acquisition cases may by more complicated as a simple proportionality and should take into account particular periods of intensive field works as well as the possible role of heavy rains promoting infection via formation of aerosols^7^.

We find that the observed seasonal patterns of dynamics in models are the result of interplay between the variation of the temperature, UV radiation and the nutrient supply level. Our models also predict highly variable daily oscillations of densities of phage-free, lytic, lysogenic bacteria, whereas the overall number of bacteria may remain fairly constant (Fig. 4C). There is also a strong dependence of resultant dynamics on the nutrients content of the environment which is described by the carrying capacity *C*. We observe that enrichment of the environment (e.g. by heavily using agricultural fertilises or using rice fields as temporal fish farms) may result in outbreaks of high bacterial numbers during warm seasons. In this case, the regular daily rhythm of variation of species densities becomes perturbed: a few days characterised by very low densities of bacteria would follow by the periods of high bacterial densities (Fig. 5C).

In our study we consider two different modelling frameworks: ODE-based and DDE-based. Both models predict similar results in terms of seasonal dynamics of bacteria-phage interactions as well as regarding the dependence of patterns on the key model parameters *C*, *b* and *K*. It this well known that biological models can be very sensitive to the choice of terms in the equations and the model structure^47–49^. Thus it is quite impressive that both models provide similar results which demonstrates the robustness of our modelling approach and strengthen theoretical predictions. On the other hand, some modification of models, for example, considering $\bar{\alpha }(T)=\alpha (T)$ would modify some of our results obtained with models (1)-(2). In particular, we found the possibility of a complete eradication of phage-free bacteria. A more thorough investigation of the model robustness towards structural changes would be needed.

Our modelling approach may suggest few possible directions towards direct testing of our main hypothesis that phages are the main control agent of bacteria. Note that the possibility of a reduction of *B. pseudomallei* by phages in soil has been demonstrated in a recent study^10^, and it is important now to verify the daily and seasonal *B. pseudomallei* population dynamics in natural systems. It follows from our model that excluding phages from the system will result in ceasing large amplitude oscillations of densities of bacteria and phages due to host-pathogen cycles and this can be easily checked empirically. The presence of pronounced variation of density of phage-free bacteria on the scale of the day or several days might indicate a strong control of *B. pseudomallei* by its phages. Another important indicator of the phage control would be measuring the ratio between the bacterial and phage numbers in the field. It is however important to note that such field studies should preferably be performed using molecular methods of detection such as qPCR as traditionally used plating techniques could under-estimate bacterial prevalence in the soil due to the presence of temperature-dependent phages^10^.

Here we build a parsimonious model of bacteria-phage interactions in a well-mixed environment as the stagnant water in the rice field. A more accurate model would include considering bacteria in the soil. In this case the distribution of environmental factors such as temperature, nutrients, mortality of phages will be highly heterogeneous and this might potentially dampen high amplitude oscillations host-pathogen observed in homogeneous environment as it happens in some predator-prey models^43,50^. This will be addressed in our future research.

Finally, our results indicate possible directions for disease management and monitoring of *B. pseudomallei*. In particular, one can estimate the risk of disease acquisition (determined by *S*) across seasons. In the case the environment does not allow a large number of bacteria (which signifies low values of carrying capacity *C*), the risky period coincides with high level of UV radiation, which causes mortality of phages (Fig. 4A). Under this scenario, the practical recommendation would be to avoid agricultural activities or use protective footwear and gloves when working in the field in the evenings (e.g. 9 pm) when phage-free bacteria amplified after the day (see Fig. 4C). In the case of nutrient rich environment - which can be a result of extensive use of fertilises or fish farming – the recommendations would be different. Under a large degree of eutrophication, one should expect high density outbreaks of phage-free bacteria during warm seasons. The duration of such outbreaks can be from several days to a month characterised by high densities of *S* regardless the time of the day. Thus, a better monitoring of disease under intensive use of fertilises is strongly recommended to report the start of the outbreak. Finally, our numerical experiments with a high background mortality rate *μ*_*c*_ of phages shows that this would cause an increase of bacterial density *S* thus enhancing the corresponding risk of disease acquisition. This has a direct application to disease management and control. Indeed, it was reported that some copper-based agrochemicals can inactivate phages thus impeding biocontrol^51^. Thus our study suggests that using such agrochemicals which are harmful for phages would amplify the risk of disease acquisition and should be implemented with a great care.

Our finding highlight that the other environmental factors that may influence phage-bacteria interactions in the rice-field ecosystems may turn to be significant for melioidosis epidemiology. For example, in North-East Thailand the salty soils are presently distributed in complex patchy pattern. So the actual hydrochemical parameters of the water of different areas should be collected in the field study, modelled in the laboratory and experimentally assessed for their influence over bacteriophage multiplication. The incorporation of this parameter into our model will increase its predictive power. Our results also indicate that fine bacteriophage biogeography data (i.e. comparative analysis of quantitative life cycle parameters of phages isolated from different locations) may be of significant value for understanding of the ecology of *B. pseudomallei* and its viruses in the environment.

## Electronic supplementary material

Supplementary material (SM)
