# Supplementary material for: Temperature-dependent virus lifecycle choices may reveal and predict facets of the biology of opportunistic pathogenic bacteria
Source: Sci Rep. 2018 Jun 25;8:9642. doi: 10.1038/s41598-018-27716-3 (PMC6018541; doi:10.1038/s41598-018-27716-3)
Supplement: Supplementary file 1 — Supplementary material (SM) [file 41598_2018_27716_MOESM1_ESM.docx]

**Supplementary Materials (SM) for the manuscript ‘Temperature-dependent virus lifecycle choices may reveal and predict facets of the biology of opportunistic pathogenic bacteria’**

Halil I. Egilmez^1^, Andrew Yu. Morozov^1^, Martha R. J. Clokie^2^, Jinyu Shan^2^, Andrey Letarov^3^ and Edouard E. Galyov^2^

In the supplementary material we provide details on experimental work to estimate the switching temperature between the lysogonenic and lytic types of infection (**SM1**), adsorption kinetic of phages (**SM2**), estimating mortality of phages and bacteria caused by UV solar radiation (**SM3**), historical UV index in Thailand (**SM4**) and also extra model simulations of bacteria-phage dynamics which are not included in the main text (**SM5**).

**Short table of Contents**

Estimating the lysogonenic-lytic switch temperature (SM1)…….…….1

Finding adsorption constant of phages (SM2) .………………………..2

Estimating UV-caused mortality rate (SM3)…………..………………3

Historical UV index in Thailand (SM4)………………………………..4

Model simulations of seasonal bacteria-phage dynamics (SM5)……....5

**SM1**

Materials and Methods: ØBp-AMP1 was isolated from our previous work, and was propagated against *Burkholderia thailandensis* E264 to prepare phage stocks (Shan et al., 2014). The plaque assay procedure of ØBp-AMP1 against E264 was carried out as described before (Shan et al., 2014), except that the plaque assay plates were incubated overnight or until individual plaques were visible at 25, 27, 29, 31, 33, 35, 37, 39, and 41 °C, respectively. The number of plaques were counted, and the resulting titres at different temperatures were calculated as PFU/ml (average of two biological replicas, each biological replica has three technical repeats).

Results: The same ØBp-AMP1 stock showed different ability to form plaques on the same E264 strain under different temperatures. The results are shown in Fig.2(B) of the main text. From 25 to 29°C, only limited number of plaques can be observed on E264 lawns, which translated into a titre of ~10^6^ PFU/ml. While when the temperature increased to 31°C, there was markedly more plaques appeared on the lawn of E264, which resulted in a significant two log increase in the phage titre to ~10^8^ PFU/ml. The phage titres increased alongside the temperate increase, such as 10^9^ PFU/ml at 33 °C and 10^10^ PFU/ml at 37°C. From 39°C onwards, the phage titres showed a slight decrease at 39°C and 41°C. This has probably been caused by alteration of the bacterial growth rate at higher temperatures. In this study, we assume that the mode of infection of phages (i.e. lysogenic or lytic) does not change at higher temperatures (i.e. *T*>40°C) and remains lytic.

Overall, the experiment clearly demonstrates that ambient temperature plays an important role in determining phage’s lytic activity. It seems that most of the phages follow lysognic life cycle rather than lytic cycle when the temperature is below 31°C, while more phages follow lytic life cycle when temperature increased to beyond 31°C.

**SM2**

The adsorption kinetics of bacteriophage Bp_AMP1.The bacteriophage BP_AMP1 was propagated on the culture of the surrogate host *Burkholderia thailandensis* E264 as it described earlier (Shan et al. 2014). For adsorption experiment the overnight culture of E264 strain was grown in LB medium depleted of NaCl (Tryptone 10g, Yeast extract 5 g, deionized water up to 1L). This culture was diluted 40 times with the same medium and grown at 37^o^C with agitation at 220 rpm for 5 h. The Cells were pelleted by centrifugation in a table-top Eppendorf centrifuge at 3000 g for 3 min and resuspended in standard LB diluted by sterile water 10 fold (the medium thus contained 1g of Tryptone, 0.5g of yeast extract and 1 g of NaCl per 1L). The optical density of the suspension was adjusted to 0.3 that corresponded to 10^8^ CFU mL^-1^ viable cells titer. The cells were allowed to adapt to the new medium at 37 ^o^C for 30 min, then the phage was added up to the concentration of 2x10^5^ PFU ml^-1^ and the mixture was incubated at 37 ^o^C. At different time points 10 µl aliquots were collected and diluted in 990 µl of the same medium to slow down the adsorption. The diluted samples were then centrifuged 5 min in the table-top centrifuge at maximum speed to spin down the cells and the phage particles attached to them. In order to determine the point “0” value the same amount of the phage was diluted in the same amount of the medium without bacterial cells and then processed as the other samples. 100 µl aliquots of the supernatants were plated by conventional double-layer method to count the free phage particles. Standard LB with 10 g of NaCl per 1L was used for plates and top agar (supplemented with 15 or 6 g of bacto-agar respectively). The plates were incubated at 37 ^o^C. The results of the experiment are presented in Fig.2C (main text) which shows the drop of the initial bacterial density within the first 30 minutes.

Phage adsorption rate to bacteria is also calculated by using the following equation

$\begin{aligned} \frac{dP}{dt}=-KNP \end{aligned}\Rightarrow\ln\left( \frac{P}{P_{0}} \right)=-KNt$,

where$N=N_{0}=1.5\times{10}^{8}$ $cells/ml^{-1}$. The regression curve (plotted via GraphPad Prism software) gives the adsorption constant *K*=$30.7$ ±$5.7\times{10}^{-8}$ $\mathrm{ml}^{-1}\mathrm{day}^{-1}$ (*R^2^*=0.84) as we converted the time units from minutes (shown in the figure) to days.

**SM3**

It is reported that $95 \%$ phages (or bacteria) go die at highest UV index and $50 \%$ phages die at lowest UV index within day in Thailand (Sinton et al. 2002). The mortality rate in day is as follow:

$\begin{aligned} &\frac{dP}{dt}=-\mu P \\ \Rightarrow& P\left( t \right)=P\left( t_{0}=0 \right)\exp\left( -\mu t \right) \\ \Rightarrow& \mu=-\ln\left( \frac{P}{P\left( t_{0}=0 \right)} \right)\frac{1}{t} \end{aligned}$,

where $t=12$ hours $=\frac{1}{2}$ day; $\frac{P}{P\left( t_{0}=0 \right)}=\frac{5}{100}=\frac{1}{20}$ for 95% of death in phages; and $\frac{P}{P\left( t_{0}=0 \right)}=\frac{50}{100}=\frac{1}{2}$ for $50\%$ of death in phages. Therefore, mortality rate of phages $\mu$ is between $5.99$ 1/day and $1.39$ 1/day due to the influence of UV. We assume that in Thailand the summer time corresponds to the UV index equal *u*=12 whereas the winter time would correspond to *u*=8. This is actually in good agreement with the historical data (see Fig.1S from SM4). Thus by neglecting the background mortality in experiments of Sinton et al., (2002), we can estimate the values of $Y_{0}=0.0746$ day ^-1^ and $k=0.366$.

**SM4**

Here we present historical UV index in the two considered provinces in Thailand.

| (A) |
| --- |
| 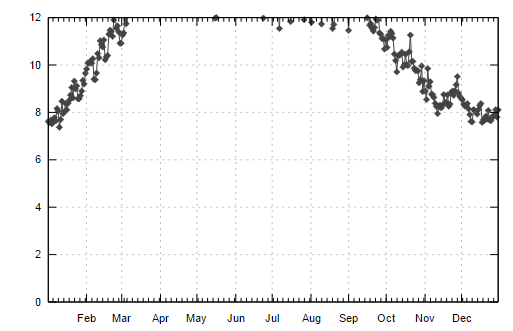 |
| 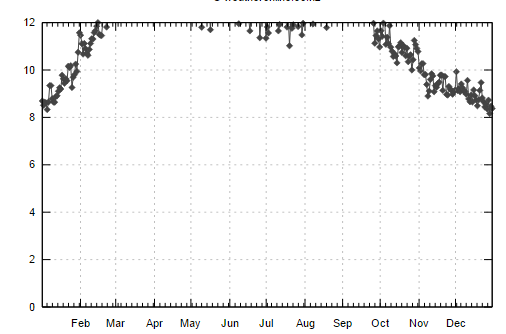  (B) |

Figure 1S: Historical UV index in 2016 for endemic two provinces in Thailand: (A) Nakhon Phanom and (B) Sa Kaeo obtained via the website: <http://www.weatheronline.co.nz/weather/maps/city>.

**SM5**

Here, in Figs. 2S-6S we show the seasonal dynamics of phages and bacteria obtained based on Model I and Model II which are not included in the main text. The details about the model and the particular province is explained each the corresponding figure caption.

(A)

|  |
| --- |
|  |

density phage/ml

(B)

**

(C)

Figure 2S: Seasonal and daily dynamics of the bacteria-phage system predicted by Model I in Sa Kaeo Province, Thailand. Seasonal variation of free phage density (CFU/ml) (A), free-phage bacteria, lysogenic bacteria and bacteria in the lytic state (B). Daily variation of phage (C) and bacterial components of the system corresponding on April 1st. The model parameters are taken from Table 1. as default values.

| **  density phage/ ml  (A) | |  | |  |
| --- | --- | --- | --- | --- |
| **  (B) | |  | |  |
|  | (D) |  |  |  |

(C)

density phage/ml

Figure 3S: Seasonal and daily dynamics of the bacteria-phage system predicted by Model II in Nakhon Phanom, Thailand. (A) Seasonal variation of free phage density (CFU/ml). (B) Seasonal variation of free-phage bacteria, lysogenic bacteria and bacteria in the lytic state. Daily variation of phage (C) and bacterial (D) components of the system corresponding on April 1^st^. The model parameters are taken from Table 1 as default values

| (A) |  |  |
| --- | --- | --- |
|  |  |  |
|  |  |  |
|   (B) |  |  |

(C)

Figure 4S: Daily average densities of phages (phage/ml) and bacteria (cell/ml) in Model I plotted for different carrying capacity in Nakhon Phanom Province. (A): $C=2\times{10}^{6}$, (B):$C=8\times{10}^{6}$, (C):$C=1\times{10}^{8}$.

|   (A) |   (B) |
| --- | --- |
|  |   (D) |
|   (E) |   (F) |

(C)

Figure 5S. Variation of daily average densities of phages (phage/ml) and bacteria (cell/ml) across the year for different values of the carrying capacity *C* (Sa Kaeo Province): (A-B) 𝐶 = 2 × 10^6^ cell/ml (C-D) 𝐶 = 8 × 10^6^ cell/ml, (E-F) 𝐶 = 1 × 10^8^ cell/ml. Simulations are based on Model I, the other parameters are taken from Table 1 as default values.

|   (A) | |
| --- | --- |
|   (B) | |
|  |  |
|  |  |

Figure 6S: Oscillations in phage density (phage/ml) and bacterial densities (cell/ml) predicted by Model I (Nakhon Phanom) between day 470 and 480 for the carrying capacity $C$ = $8\times{10}^{6}$.

**SM6**

Here, in Figs.7S-12S we show the seasonal dynamics of phages and bacteria obtained based on Model I in the Nakhon Phanom Province in the case where either the temperature or the UV- solar radiation level is kept constant whereas the other factor is being varied according to the historic data.

(A)

(B)

Figure 7S. Variation of daily average densities of phages (phage/ml) and bacteria (cell/ml) across the year for a constant value of temperature (Nakhon Phanom); the UV level is variable according to the historic data. Here *T*=39^0^C which represent a high temperature regime. Simulations are based on Model I, the carrying capacity 𝐶 = 2 × 10^6^ cell/ml; the other parameters are taken from Table 1 as default values.

(A)

(B)

Figure 8S. Variation of daily average densities of phages (phage/ml) and bacteria (cell/ml) across the year for a constant value of temperature (Nakhon Phanom); the UV level is variable according to the historic data. Here *T*=20^0^C which represent a low temperature regime. Simulations are based on Model I, the carrying capacity 𝐶 = 2 × 10^6^ cell/ml; the other parameters are taken from Table 1 as default values

(A)

(B)

Figure 9S. Variation of daily average densities of phages (phage/ml) and bacteria (cell/ml) across the year for a constant value of temperature (Nakhon Phanom); the UV level is variable according to the historic data. Here *T*=29.1^0^C which represent the average over the year. Simulations are based on Model I, the carrying capacity 𝐶 = 2 × 10^6^ cell/ml; the other parameters are taken from Table 1 as default values

(A)

(B)

Figure 10S. Variation of daily average densities of phages (phage/ml) and bacteria (cell/ml) across the year for a constant level of UV solar radiation (Nakhon Phanom); the temperature is variable according to the historic data. Here *u*=12 which represent the highest UV index. Simulations are based on Model I, the carrying capacity 𝐶 = 2 × 10^6^ cell/ml; the other parameters are taken from Table 1 as default values.

(A)

(B)

Figure 11S. Variation of daily average densities of phages (phage/ml) and bacteria (cell/ml) across the year for a constant level of UV solar radiation (Nakhon Phanom); the temperature is variable according to the historic data. Here *u*=0 which represent the lowest UV index. Simulations are based on Model I, the carrying capacity 𝐶 = 2 × 10^6^ cell/ml; the other parameters are taken from Table 1 as default values.

(A)

(B)

Figure 12S. Variation of daily average densities of phages (phage/ml) and bacteria (cell/ml) across the year for a constant level of UV solar radiation (Nakhon Phanom); the temperature is variable according to the historic data. Here *u*=10.7 which represent the average over the year UV index. Simulations are based on Model I, the carrying capacity 𝐶 = 2 × 10^6^ cell/ml; the other parameters are taken from Table 1 as default values.
